# Food Allergenicity Evaluation Methods: Classification, Principle, and Applications

**DOI:** 10.3390/foods14122005

**Published:** 2025-06-06

**Authors:** Huiqiao Zhou, Xiao Chen, Xin Li

**Affiliations:** 1 State Key Laboratory of Food Science and Resources, Nanchang University, Nanchang 330047, China; 1220012511@student.must.edu.mo (H.Z.); chenxiao6141@email.ncu.edu.cn (X.C.); 2 School of Medicine, Macau University of Science and Technology, Macau 999078, China; 3 School of Food Science and Technology, Nanchang University, Nanchang 330047, China; 4 Jiangxi Province Key Laboratory of Food Allergy, Nanchang University, Nanchang 330047, China

**Keywords:** food allergy, food allergen, allergenicity evaluation, epitope, immune responses, IgE-mediated allergy

## Abstract

The incidence of food allergies is increasing annually, emerging as a significant global concern for food safety and public health. To prevent allergic reactions, patients are advised to avoid ingesting allergenic foods. However, completely avoiding contact with such foods in real life is often challenging. Therefore, the development of reliable allergenicity evaluation methods is essential to assist food allergy patients in avoiding exposure to allergenic foods. These evaluation methods include mass spectrometry-based methods, bioinformatics predictions, simulated digestion, enzyme-linked immunosorbent assays, cell-based models, and animal models. Each method operates on distinct principles and is suited for specific evaluation contexts. This review systematically summarizes the pathogenesis of food allergies and details the principles and practical applications of common allergenicity evaluation methods. By highlighting recent studies in food allergenicity evaluation, we aim to deepen the understanding of allergenicity assessment and offer an overview with perspectives on its enhancement.

## 1. Introduction

Food allergy is defined as a pathological immune response triggered by the ingestion of specific foods, which can affect multiple organs, including the gastrointestinal tract, skin, and respiratory system [1,2]. Approximately 90% of food allergies are attributable to proteins present in certain foods, with common allergenic sources including milk, eggs, peanuts, tree nuts, fish, shellfish, and wheat [1]. The rapid development of logistics and the globalization of food supply chains have significantly increased the variety of foods available; however, this diversification has also been associated with a consistent rise in the incidence of food allergies. A cross-sectional survey on the prevalence of food allergies among children under 18 years of age in the United States reported a prevalence rate of 7.6% in 2018, a notable increase from 3.9% in 2007 and 3.3% in 1997 [3,4]. Similarly, a report from the United Kingdom documented a rapid rise in food allergy prevalence among children under 15 years of age, reflecting an annual increase of 6.6% from 1998 to 2018 [5]. In China, epidemiological data from the Chongqing province indicated that the prevalence of food allergies among children rose from 3.5% in 1999 to 7.7% in 2009, and further to 11.1% in 2019 [6].

Currently, there is no definitive “cure” for food allergies. For individuals with mild symptoms, antihistamines and corticosteroids can be administered judiciously to alleviate allergic reactions [7]. In contrast, patients experiencing severe symptoms require epinephrine in addition to antihistamines and corticosteroids for both prevention and treatment [7]. Despite these emergency measures, strict avoidance of allergenic foods remains essential for preventing allergic reactions in both mild and severe cases. Consequently, the evaluation of allergenicity in food components and the labeling of potential allergens in food products have become crucial strategies to prevent accidental ingestion of allergenic foods by sensitized individuals, and to mitigate the risk of allergic reactions [8]. To date, several methods have been developed for evaluating the allergenicity of food components, including bioinformatics-based predictions, mass spectrometry, in vivo- and in vitro-simulated digestion, enzyme-linked immunosorbent assays (ELISA), cell models, and animal models. Each method operates on distinct principles and focuses on different aspects of allergenicity evaluation. Although numerous studies have been published regarding the evaluation of food allergenicity, a comprehensive review of the methods employed for this purpose is currently lacking.

As previously described, this review aims to summarize the mechanisms underlying food allergies and the fundamental principles of food allergenicity assessment methods. Additionally, we provide a comprehensive overview of recent applications and advancements in these allergenicity assessment techniques for food allergens. This review aims to provide new perspectives and inspire further research and applications in the evaluation of food allergenicity.

## 2. The Mechanisms Underlying Food Allergy Development

Food allergies can be classified into three subtypes based on the involvement of allergen-specific IgE antibodies: IgE-mediated food allergy, non-IgE-mediated food allergy, and mixed-mediated food allergy. Among these, the pathogenesis of non-IgE-mediated and mixed-mediated food allergies remains less well-defined. Potential mechanisms may involve an imbalance of T cells and related cytokines [9,10,11], overactivation of granulocytes [12,13], and dysfunction of immune tolerance [14]. In contrast, the mechanisms underlying IgE-mediated food allergy are comparatively well-established and will be systematically presented later. Furthermore, the fundamental principle of allergenicity evaluation is constructed based largely on allergenic epitopes involved in IgE-mediated responses. Accordingly, existing methods for assessing food allergenicity predominantly rely on these IgE-associated principles [15,16]. Therefore, this review will focus on summarizing the mechanisms underlying IgE-mediated food allergies.

### 2.1. Allergenic Epitope and Food Allergy

Allergenic epitopes serve as the structural basis for the binding of food allergens to antibodies and are the primary initiators of food allergic reactions. Based on their spatial structures, allergenic epitopes can be classified into linear and conformational epitopes [17,18]. Linear epitopes consist of sequentially arranged amino acid residues, whereas conformational epitopes are formed by discontinuous amino acid residues that are spatially adjacent. Furthermore, allergenic epitopes can also be categorized based on the types of receptor cells they bind to, distinguishing between B-cell epitopes and T-cell epitopes [18,19]. B-cell epitopes encompass both linear and conformational types, while T-cell epitopes are predominantly linear. Among B-cell epitopes, linear epitopes that bind to IgE and IgG are the major sensitizing epitopes involved in allergic reactions [18,20]. Following digestion and absorption, if allergenic epitopes from food allergens remain intact and are recognized by the immune system, they may trigger food allergic reactions [21].

### 2.2. Digestion and Absorption of Allergens

Food allergens are predominantly proteins. Upon entering the digestive system, the gastrointestinal enzymatic cleavage disrupts the secondary and tertiary structures of food allergen proteins, resulting in their degradation into low-allergenicity peptides [21,22]. This process effectively helps prevent the induction of an allergic immune response in the host [21,22]. However, food processing techniques such as thermal treatment, irradiation, and chemical processing, as well as modifications involving enzymes and bioactive substances, can alter the digestibility of food allergens. These changes may inhibit complete enzymatic degradation, leading to the retention of food components containing allergenic epitopes and consequently increasing the risk of triggering allergic immune responses in the host [21,23,24,25].

**Figure 1:**
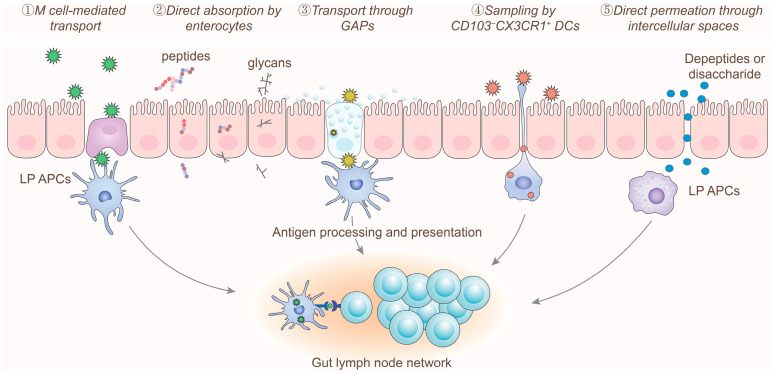
Mechanisms for food allergen delivery. Allergens can migrate from the intestinal lumen to the subepithelial layer through various pathways: large soluble proteins mainly utilize M cell-mediated transcytosis and goblet cell-associated antigen passages (GAPs), or they are captured by CD103^−^CXCR1^+^ dendritic cells (DCs) located beneath the epithelial layer. Conversely, small-molecular-weight particles can directly enter enterocytes via absorption or permeate through intercellular spaces. Abbreviation: LP: lamina propria; APCs: antigen-presenting cells.

After digestion and degradation, food allergens that reach the small intestine can be recognized and presented to the immune system through several mechanisms (Figure 1) [26,27,28]: (1) M cell-mediated transport: Soluble protein aggregates are initially processed, absorbed, and transported by M cells located in the follicle-associated epithelium. Subsequently, these antigens are recognized and presented to the immune system by antigen-presenting cells (APCs) within the lymphoid follicles. (2) Direct absorption by enterocytes: Amino acids, oligopeptides, and glycans with molecular weights less than 1500 Da can be directly absorbed and utilized by enterocytes for transport. (3) Goblet cell-associated antigen passage: Soluble protein particles and peptides can be absorbed and transported to the basal layer via GAPs. These antigens are then recognized, processed, and presented to naïve T cells and antigen-specific regulatory T cells (Tregs) by CD103^+^ DCs, which are essential for maintaining the immune activity of intestinal Tregs [29,30]. (4) Sampling by CD103^−^CX3CR1^+^ DCs: CD103^−^CX3CR1^+^ DCs located beneath the intestinal epithelium form trans-epithelial dendrites that actively sample low molecular weight protein particles and microbial antigens from the intestinal lumen, thereby maintaining tolerance to commensal bacteria and certain water-soluble antigens [31]. (5) Direct permeation through intercellular spaces: Some amino acids and peptides with molecular weights less than 600 Da can directly permeate through intercellular spaces into the subepithelial region, from where they are transported by blood vessels and lymphatic vessels.

Under physiological conditions, large food allergens that retain their allergenic epitopes are primarily transported through M cells and GAPs to induce immune tolerance [26,27,28]. During this process, endogenous components such as mucins, antimicrobial peptides, and secondary bile acids can be co-transported with allergenic proteins, which further diminishes their sensitizing potential [32,33]. In contrast, smaller molecular allergens that can be absorbed by enterocytes or permeate through intercellular spaces exhibit lower allergenicity and are less likely to provoke an allergic immune response in the host [28,34]. Therefore, various lifestyle factors, including high-fat diets, alcohol consumption, irregular sleep patterns, the use of antibiotics, and intestinal inflammation or infection, can compromise the integrity of the intestinal barrier. This disruption increases the likelihood of leakage of large molecular food allergens across the intestinal barrier, thereby elevating the risk of developing food allergies [35,36,37].

### 2.3. Immune Responses to Allergens

**Figure 2:**
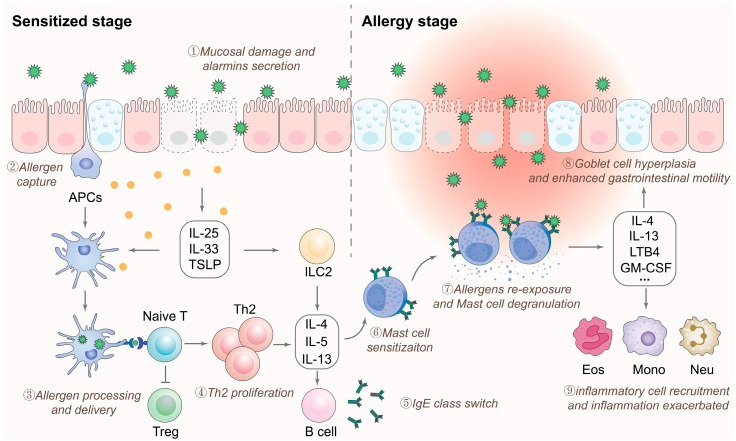
Mechanisms of IgE-mediated food allergy. Damage to the intestinal epithelial barrier upon exposure to food allergens allows allergen penetration and induces the release of alarmins. This activates antigen-presenting cells (APCs) and group 2 innate lymphoid cells (ILC2s), promoting naïve T cell differentiation into Th2 cells. Further, Th2 cytokines from ILC2s and Th2 cells induce IgE class switching in B cells. Allergen-specific IgE then binds to FcεRI receptors on mast cells, leading to mast cell sensitization. Upon re-exposure to the same allergens, sensitized mast cells can rapidly recognize the incoming allergens via IgE antibodies and initiate the degranulation process, releasing cytokines and chemokines that drive epithelial hyperplasia, inflammatory cell recruitment, and allergic inflammation. Abbreviations: Eos: eosinophils; Mono: monocytes; Neu: neutrophils; Treg: regulatory T cell; TSLP: thymic stromal lymphopoietin; GM-CSF: granulocyte–macrophage colony-stimulating factor; LTB4: leukotriene B4.

Following translocation across the intestinal barrier, food allergens that retain their allergenic epitopes can be recognized by the immune system. This recognition results in the release of bioactive mediators from effector cells, which can induce tissue damage and lead to clinical symptoms. The progression of food allergies can be divided into two phases: the sensitization phase and the effector phase (Figure 2) [38,39,40].

(1) Sensitization phase: Upon entering the host, allergens penetrate the compromised intestinal epithelial barrier and are subsequently captured and processed by APCs, such as macrophages and DCs. Concurrently, in response to epithelial-derived alarmins, including IL-25, IL-33, and thymic stromal lymphopoietin (TSLP), APCs upregulate the expression of OX40 ligand. Meanwhile, group 2 innate lymphoid cells (ILC2) increase the secretion of type 2 cytokines, such as IL-4, IL-5, and IL-13. These factors collectively promote the differentiation of type 2 helper T cells (Th2 cells). Subsequently, activated Th2 cells produce cytokines such as IL-4, IL-5, IL-9, and IL-13, which induce specific B cells to undergo class switching to IgE-secreting plasma cells, resulting in the generation of large amounts of allergen-specific IgE antibodies. These IgE antibodies then attach to the high-affinity IgE receptor (FcεRI) on the surfaces of mast cells and basophils via their Fc regions, thereby sensitizing the host.

(2) Effector phase: Upon re-exposure of a sensitized host to the same allergen, the allergen binds to allergen-specific IgE receptors on the surface of mast cells and basophils, leading to the cross-linking and clustering of multiple FcεRI receptors. This interaction transmits activation signals intracellularly through the β and γ chains of the FcεRI receptor, engaging downstream signaling pathways that activate the cells. The activation results in the release of preformed and newly synthesized bioactive mediators, including histamine, serotonin, leukotrienes, platelet-activating factor, tryptase, heparin, and prostaglandin D2, which further leads to the manifestation of various allergic symptoms.

## 3. Assessment Methods for the Allergenicity of Food Components

**Figure 3:**
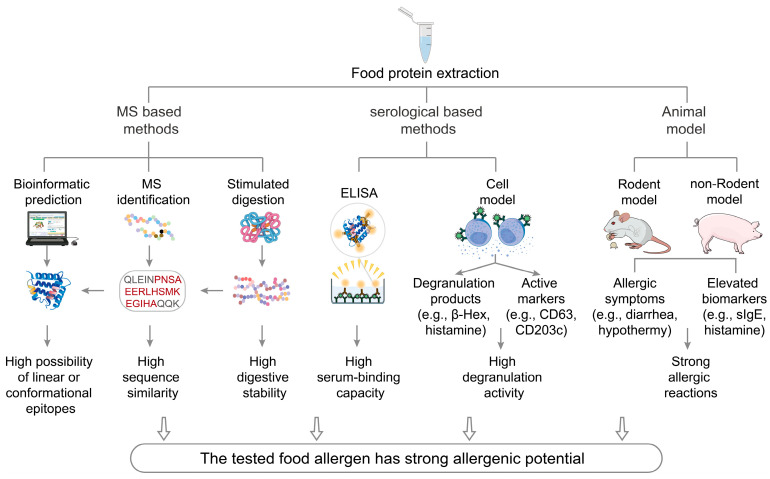
The classification and framework of allergenicity assessment methods. Allergenicity assessment methods can be classified into three categories: MS-based methods (e.g., MS identification, bioinformatic prediction, and simulated digestion), serology-based methods (e.g., ELISA and cell models), and animal-based methods (e.g., rodent and non-rodent models). MS-based methods detect allergen sequences to predict allergic epitopes. Serology-based methods rely on antigen–antibody reactions and depend heavily on the availability of allergen-specific sera. Animal-based models replicate sensitization routes to generate in vivo data reflecting allergenicity and sensitization mechanisms similar to those in humans. Each positive result indicates that the tested food allergen has the potential to induce an allergic reaction. Abbreviations: β-Hex; sIgE: allergen-specific IgE.

As previously described, food allergens undergo a series of enzymatic digestion and trans-epithelial transport processes before being recognized by the immune system. The likelihood of triggering an allergic immune response is influenced by the molecular structure of residual allergens and the nature and quantity of allergenic epitopes [18,20,21]. Similarly, various assessment methods have been developed to evaluate the allergenicity of food allergens by mimicking the immune presentation processes (Figure 3). The principles and recent advancements of these assessment methods are summarized in Table 1.

### 3.1. Mass Spectrometry-Based Methods

The fundamental principle of mass spectrometry (MS) involves ionizing a sample to generate molecules or molecular fragments with varying charges. These ions are then accelerated by an electric field and directed into a mass analyzer, where they are separated based on differences in their mass-to-charge ratios under the combined influence of electric and magnetic fields. The resulting MS analysis produces characteristic peptide fragments or fingerprint spectra. By comparing these characteristic data with information from established protein databases, accurate identification of protein types and allergenic epitopes can be achieved [41,42]. Historically, the application of early MS techniques in protein and peptide analysis was somewhat limited due to the polar nature, low volatility, and thermal instability of most proteins and peptides. However, the introduction of soft ionization techniques, such as Matrix-Assisted Laser Desorption/Ionization and Electrospray Ionization, has significantly broadened the application of mass spectrometry in these fields.

The fundamental advantage of MS in evaluating food allergenicity is its capability to analyze proteins based on their amino acid sequences or peptides generated through enzymatic digestion, rather than on higher-order protein structures, which makes it an indirect method for supporting food allergenicity assessment. This characteristic allows MS to effectively mitigate the potential impacts of food processing on detection results [42,43]. Additionally, the automated and standardized workflow of MS enhances the sensitivity and stability of detection, thereby ensuring the reliability and reproducibility of the results. Currently, MS can be utilized for both qualitative and quantitative detection of food allergenic components. Qualitative detection is typically achieved through techniques such as peptide mass fingerprinting and peptide–fragment fingerprinting [41,44,45], while quantitative detection often employs methods like isotope labeling and selected reaction monitoring [41,46].

**Table 1:** Comparison of established food allergenicity evaluation methods.

Methods	Detection Principle	Advantages	Shortcomings	Applicable Range	Predictive Accuracy
Mass spectrometry [41,42,43]	Comparing the peptide segments with known epitopes that can be recognized by T/B cells	The procedure is relatively simple and allows for quick results	Large-scale instruments and a reliable database are required, and only linear epitope information can be obtained	Suitable for almost all allergenic foods	Relatively low
Bioinformatic prediction [47,48,49,50]	Comparing peptide segments with allergic epitopes and making predictions of potential linear and conformational epitopes	Computational simulation can identify a wide range of potential epitopes	This process demands proficient operational skills and a reliable database; otherwise, inaccurate results may occur	Suitable for almost all allergenic foods	Relatively low
Simulated digestion [15,21,22]	Detecting the structure, molecular weight, and allergic epitopes in residual digestion products	It can reflect the effects of different digestion conditions and interactions between food components and allergic epitopes after digestion	The operation is relatively complex, and the experimental results may not reflect the actual situation accurately	Suitable for almost all allergenic foods	Relatively low
ELISA examination [51,52,53]	Detecting the allergic epitopes based on the antigen–antibody interaction	Can quickly determine the components responsible for potential allergic reactions in patients	Obtaining suitable serum can be challenging	Suitable for allergenic foods tested with human or animal serum	Medium
Cell model [54,55]	The interaction between allergens and allergen-specific IgE can induce mast cell or basophil degranulation	Can accurately determine the presence of substances that trigger mast cell or basophil activation	Obtaining suitable serum can be challenging	Suitable for allergenic foods tested with human or animal serum	Relatively high
Animal model [56,57,58]	Sensitizing animals with allergens and observing changes in allergic symptoms and biomarkers after allergen challenge	The experimental results best capture the actual allergenic potential of the tested food components through multiple aspects	The experiment is costly, time-consuming, and may involve ethical issues	Suitable for allergenic foods tested with protein reference material	Relatively high

MS has been successfully employed to evaluate the allergenicity of food components in dairy products, shrimp, meat, and other food items (Table 2). Monaci et al. [59] utilized Liquid Chromatography-Quadrupole-Time of Flight Mass Spectrometry to assess milk allergens in thermally processed foods, successfully identifying specific peptides associated with milk allergens. These peptides can serve as markers for preliminary screening and validation of target allergens in future studies, providing a foundation for related research. Stasio et al. [60] extracted peanut components using SDS-PAGE and identified their allergenicity using a Quadrupole-Orbitrap Mass Spectrometer. In the MS spectra, the ten most intense ion peaks were selected for fragmentation analysis. The spectral data were then converted into protein identification results by integrating instrument analysis software with the NCBI database, thereby assessing the allergenic potential of the peanut proteins.

Pilolli et al. [61] developed a relatively rapid sample processing procedure involving ultrasound-assisted Size Exclusion Chromatography for extraction and purification and constructed a multidimensional chromatographic analysis system. Utilizing this system, they established an efficient and streamlined analytical workflow, successfully identifying the allergenicity from eggs, milk, soy, hazelnut, and peanut components in complex food matrices through Selected Reaction Monitoring analysis. Despite the advantages listed above, MS cannot detect conformational epitopes and heavily depends on sequence homology to known allergens. Moreover, it cannot assess the allergenic potential of novel proteins. The stringent requirements for allergen extraction and purification, combined with the high costs of instrument setup, have further limited the widespread application of MS in food allergenicity assessment.

### 3.2. Bioinformatic Prediction

Bioinformatics is an interdisciplinary field that integrates biology, mathematics, and computer science, with the goal of analyzing and interpreting vast amounts of biological data. Its application in assessing food allergenicity relies on the principle of homology analysis. Specifically, the amino acid sequence of the protein under investigation is compared against internationally recognized databases of known allergen sequences to predict the likelihood of cross-reactivity between the protein of interest and established allergens [47,48,49,50].

In bioinformatics-based allergenicity assessments, the construction of a comprehensive and robust database of known allergens is essential. Currently, Allergome is the largest global allergen database, containing over 10,000 allergen sequence entries. In addition to this resource, several specialized databases are available, including the allergen search engine AllergenOnline (http://www.allergenonline.org/, accessed on 25 May 2025), the allergen nomenclature database ALLERGEN NOMENCLATURE (https://www.allergen.org/, accessed on 25 May 2025), the allergen protein family database ALLFAM (https://www.meduniwien.ac.at/allfam/, accessed on 25 May 2025), and the allergen structure databases SDAP (https://fermi.utmb.edu/, accessed on 25 May 2025) and COMPARE (https://db.comparedatabase.org/, accessed on 25 May 2025). These databases provide extensive data support and analytical tools for allergen research, facilitating a deeper understanding of allergen characteristics and their sensitization mechanisms.

In recent years, advancements in the understanding of allergens, alongside the continuous accumulation of related data, have led to the establishment and refinement of bioinformatics technologies for predicting the allergenicity of food components. For instance, FUGUE can predict the three-dimensional structures of proteins from their sequences and identify homologous proteins with low sequence similarity through combined sequence and structural similarity analyses [62]. The Allermatch system utilizes sequence alignment techniques to predict protein allergenicity [63]. Additionally, AlgPred integrates multiple prediction tools into a unified framework, facilitating effective predictions of novel allergens and their epitopes [64].

In terms of specific applications, Hu et al. [65] utilized DNAMAN software to predict novel IgE-binding epitopes of the bovine milk allergen BLG and identified four key amino acids: A34, A37, R40, and V. Garino et al. [66] employed the Allergome database and the BLAST (version 1.3.0) sequence alignment tool in conjunction with seven different allergenicity prediction software packages. Through sequence and domain analyses, they ultimately evaluated the allergenicity of 28 allergenic lipid transfer proteins across various species.

Despite these advantages, the use of bioinformatics in predicting food allergenicity has several limitations. Firstly, the accuracy of prediction models is limited by the quality and quantity of available allergen data [47]. Incomplete or inaccurate data can lead to unreliable predictions. Furthermore, the experience of the operator may also affect the results obtained. Secondly, predicting the allergenic potential of complex mixtures or processed foods remains challenging due to the dynamic nature of protein interactions and modifications. The development of artificial intelligence and improvement of allergen databases may help overcome these limitations [67].

### 3.3. Simulated Digestion

The simulated digestion method for allergenicity assessment involves recreating in vivo digestive conditions by mimicking the physiological characteristics of humans. This approach employs relevant digestive enzyme systems to evaluate the digestive and absorption properties of samples in vitro [21,22,68]. Allergenic proteins that remain intact and immunogenic after processing and gastrointestinal digestion, and prior to entering the immune system, may possess potential allergenicity. Generally, the greater the digestive stability of a protein, the higher its allergenic potential; conversely, lower digestive stability is associated with reduced allergenicity [21,22]. Based on variations in digestive conditions, the simulated digestion method can be further categorized into two approaches: static in vitro-simulated digestion and dynamic in vitro-simulated digestion.

The static simulated digestion system can be divided into three main components based on the digestive phases: the oral phase, the gastric and small intestinal phase, and the large intestinal fermentation phase [69]. Given the rapid onset of most food allergic reactions and the distribution characteristics of the human lymphatic immune system, the evaluation of food allergenicity typically focuses on simulating the digestive processes of the mouth, stomach, and duodenum, with less emphasis on the large intestinal fermentation phase [69,70]. This approach offers several advantages, including operational simplicity and broad applicability.

In assessing the potential allergenicity of food allergens, commonly used static in vitro digestion methods are primarily based on standardized protocols proposed by international organizations, such as the United States Pharmacopeia (USP) and INFOGEST. The INFOGEST method, first introduced by Minekus et al. in 2014, aims to enhance the reproducibility and comparability of digestion results across different laboratories through standardized experimental conditions and procedures [69]. Subsequently, Brodkorb et al. optimized this method, developing the INFOGEST 2.0 in vitro static digestion protocol [71]. This updated version provides more detailed descriptions of digestive fluids, procedural steps, and equipment, as well as specific guidelines for enzyme activity assays, sample collection, and processing. Currently, the INFOGEST 2.0 method has been widely applied in studies evaluating the potential allergenicity of various food allergens [72,73,74].

Dynamic in vitro-simulated digestion represents an innovative approach that integrates a range of dynamic changes, including food intake, secretion of digestive fluids, gastrointestinal emptying, peristalsis, and absorption across the gastrointestinal mucosa. This method maximizes the simulation of physiological conditions within the human or animal gastrointestinal tract. Compared to traditional static in vitro-simulated digestion methods, dynamic approaches address their limitations by minimizing manual operations and maintaining consistent simulated digestion conditions, effectively reducing experimental errors arising from operational variability or fluctuations in digestion conditions [21,22].

Moreover, dynamic in vitro digestion models can replicate digestive conditions across various physiological or health states, including those of infants, adults, individuals with gastrointestinal diseases, allergic patients, and diabetic patients. This capability is crucial for obtaining critical data, such as the threshold levels of food allergens [21,22,69,71]. Unlike static digestion processes, dynamic conditions influence the digestive stability of proteins and their interactions with other food components, which may significantly impact the evaluation of the allergenic potential of digested products and the kinetic behavior of digestion-resistant peptides. Consequently, dynamic in vitro digestion offers a more accurate representation of food allergen digestion.

For instance, Prodic et al. investigated the allergenic potential of digestion-resistant peptides released during the simulated gastric digestion of peanuts [75]. Their study revealed that peptides with strong allergenic potential retained continuous epitope sequences, primarily originating from the 2S albumin in peanuts, findings that are closely consistent with observations in real-world research scenarios. Despite this, the limitations of simulated digestion in completely reflecting real ingestion conditions in humans limit its applications in broad food allergenicity evaluation.

**Table 2:** Applications of allergenicity evaluation methods on food components.

Methods		Food Matrix	Experimental Design	Epitope Features	Reference
Mass spectrometry-based methods	LC-MS/MS	Cookies	Establishing a fast sample-handling procedure and using the signature peptide sequences to evaluate the allergenic potential.	Linear epitope	[61]
	LC-MS/MS	Insects	Proteins extracted from cricket and black soldier fly were sequenced and their cross-reactivity to allergens from shrimps was examined.	Linear epitope	[76]
	RPLC-ESI-HRMS	Chocolate	Utilizing the signature peptide sequences to search for and align the target allergens.	Linear epitope	[77]
	LC-QQQ-MS	Meat-based foodstuffs like cooked meat and sausages	Utilizing the signature peptide and its isotope-labeled peptide as standards and internal standards, respectively.	Linear epitope	[78]
	LC-MS/MS	Shrimps	The signature peptides were confirmed and synthesized as the quantitative peptides, and the relative isotope-labeled internal standards were used in the quantitative analysis.	Linear epitope	[79]
Bioinformatic prediction	Machine learning	Microalgae	DIA-NN was used to create a peptide spectrum library from PBQC DIA-MS data, with the “Deep learning-based spectra” option enabled to predict potential allergenicity.	Linear epitope	[80]
	Database-based searching and prediction	Milk	Utilizing the UniProt protein database to predict key homology sequences and use DNAMAN software to identify allergic sequence alignments.	Linear epitope	[65]
	Database-based searching and prediction	Egg products	The linear epitope of potential allergens was predicted based on computational approaches including Immunomedicine Group, ABCpred, BCEpred, BepiPred-3.0 Server, and DNAStar.	Linear epitope	[81]
	Machine learning	Sesame	Utilizing AlphaFold2 to predict the 3D structure of potential allergens based on known sequences and evaluate the accuracy of the predicted results using Psi/Phi Ramachandran plots and the WHATCHECK Complete suite.	Conformational epitope	[82]
	Machine learning	Fruits and nuts	Peptides similar to known IgE epitopes can be identified with the property distance tool, and conformational epitopes can be identified by the cross–react method, both based on the SDAP webserver.	Conformational epitope	[83]
Simulated digestion	Static simulated digestion	Peanuts	Verifying the correlation between allergenicity and resistance to proteolysis by pepsin.	Linear epitope	[75]
	Static simulated digestion	Milk	Verifying the contribution of protein phosphorylation to food allergenicity before and after simulated digestion.	Linear epitope	[68]
	Dynamic simulated digestion	Shrimps	Examining the effects of digestion stability on food allergenicity during dynamic digestion.	Conformational epitope	[84]
	Dynamic simulated digestion	Milk	Evaluating the effects of thermal processing and lactosylation on digestibility and allergenicity.	Conformational epitope	[85]
ELISA examination	Indirect ELISA	Insects	Evaluating the risk of food allergy from eating Gryllus bimaculatus in patients allergic to shrimps, using ELISA and IgE crosslinking luciferase assays.	Conformational epitope	[86]
	Sandwich ELISA	Crustacean products	Designing a system using polyclonal and monoclonal antibodies to capture and recognize potential allergens.	Linear epitope and Conformational epitope	[87]
	Sandwich ELISA	Fish products	Polyclonal antibodies were raised against parvalbumins from 13 fish species of seven fish orders, selected for molecular diversity and immunoreactivity.	Conformational epitope	[88]
	Sandwich ELISA	Almonds	Combining sandwich ELISA with lateral flow immunoassay to identify potential allergens.	Conformational epitope	[89]
Cell model	Human peripheral blood basophils	Peanuts	Assessing diagnostic application of natural and recombinant allergens by BAT and identifying IgE-binding epitopes using oligopeptide microarrays.	Linear epitope	[90]
	KU812 cell model	Soymilk	Utilizing a sensitized cell model to evaluate the changes in spatial structure and sequences after bacterial fermentation.	Linear epitope	[91]
	LAD2 cell model	Shrimps	The LAD2 cell degranulation assay was used to assess the binding affinity and antigenicity of the allergenic epitopes.	Linear epitope	[92]
	RBL-2H3 cell model	Mealworm	The RBL-2H3 cell model was used to evaluate the allergenicity of mealworm tropomyosin in comparison with shrimp and chicken tropomyosin.	Conformational epitope	[93]
Animal model	BALB/c mice model	Soybean meal	Utilizing the BALB/c mice model to determine whether solid-state fermentation could degrade major allergens and reduce the potential allergenicity of soybean meal.	Linear and conformational epitope	[94]
	C3H/HeNCrl mice model	Milk	Using the C3H/HeNCrl mice model to examine the susceptibility of potential allergens.	Conformational epitope	[95]
	BN rat model	Egg and potato components	Utilizing the BN rat model to determine the potential allergenicity of novel proteins in genetically modified food.	Conformational epitope	[96]
	Pig model	Soybean products	Using the pig model to evaluate the allergenicity of soybean protein after deglycosylation and pepsin digestion.	Linear and conformational epitope	[97]
	Monkey model	Transgenic rice	Evaluating the allergenicity of transgenic crops by examining the hematological, biochemical, pathological, and histopathological changes in sensitized monkeys.	Linear epitope	[98]

### 3.4. ELISA

This method is based on the specific antigen-antibody reaction and primarily evaluates the allergenic potential of target components by measuring their binding to specific IgE in the serum of allergic patients [51,52]. ELISA is commonly used to detect specific epitopes in food components, thereby reflecting the structural integrity of antigens, but it does not directly measure allergenicity. Given the limited availability of serum from allergic individuals, researchers often use specific IgE serum derived from immunized animals as an alternative for serological experiments.

The ELISA assay is one of the most widely used methods for evaluating food allergenicity, with a variety of commercially available ELISA kits on the market. Common types of ELISA include sandwich ELISA, competitive/inhibition ELISA, direct ELISA, and indirect ELISA, each characterized by distinct features and suitability for different testing conditions [53].

Sandwich ELISA is particularly effective for analyzing complex samples, as it does not require purification of the target antigen prior to measurement, while still achieving high sensitivity and specificity. Competitive/inhibition ELISA is suitable for detecting small molecular weight antigens, measuring the concentration of antigens or antibodies in the sample by analyzing interference with the expected signal output. Direct ELISA, which omits the use of a secondary antibody and the signal amplification step, is appropriate for analyzing immune responses to antigens, but has relatively lower detection sensitivity. Indirect ELISA involves coating the antigen onto a microplate, where the unlabeled primary antibody binds to the specific antigen during detection, followed by the application of an enzyme-conjugated secondary antibody specific to the host species of the primary antibody, thereby completing the measurement of the target [53,99,100].

This far, numerous studies have reported the use of ELISA technology to assess the allergenicity of food components. For instance, He et al. developed several ELISA-based detection methods for evaluating the allergenic potential of components such as ALA, BLG, and casein in food matrices [86,101,102]. Similarly, Arun et al. employed sandwich ELISA to assess the allergenic potential of peanut allergens, including Ara h 1, Ara h 2, Ara h 3, Ara h 6, and Ara h 8, in food matrices [103]. A substantial number of application examples have been well summarized in the literature [104], and thus will not be reiterated here.

### 3.5. Cell Model

In recent years, cell culture technology has been widely applied in molecular biology, genetics, immunology, and other fields, evolving into a significant biotechnological tool with notable achievements [54]. In the evaluation of food allergenicity, mast cells and basophils are the most commonly used cellular models, primarily due to their critical roles as effector cells in allergic reactions [54,55,105]. Upon activation, mast cells can induce bronchial spasms and increase vascular permeability, leading to clinical symptoms by synthesizing and releasing various mediators, such as histamine and leukotrienes [55]. Additionally, mast cells release multiple cytokines that further promote the development and progression of allergic diseases [105]. Therefore, by co-incubating the food allergenic components under investigation with sensitized mast cells or basophils, and detecting the levels of effector molecules in the culture system, researchers can assess the allergenic potential of the test food components [54]. These methods offer a controllable, stable, and reproducible platform for investigating allergic reactions. Their use enables efficient screening of food allergens and evaluation of their sensitizing potential in an ethically sound and practically advantageous manner compared to in vivo experiments. Consequently, these methods hold significant value and promise for predicting human allergic responses in food allergenicity assessment.

Currently, commonly used cell lines for food allergenicity assessment include Rat Basophilic Leukemia 2H3 (RBL-2H3) and human mast cell lines, such as Human Mast Cell Line 1 (HMC-1) and Laboratory of Allergic Diseases 2 (LAD2) [54,106]. Additionally, there are reports of using bone marrow-derived mast cells for evaluation. However, these cells require 4 to 6 weeks to mature. The prolonged culture period, high material consumption, and associated costs limit their application in food allergen detection [107]. Consequently, commercially available cell lines like RBL-2H3, HMC-1, and LAD2 are more frequently employed for evaluating food allergenicity. For example, Jiang et al. [108] successfully identified the major fish allergen parvalbumin by detecting fluorescence signals in RBL-2H3 cells. Similarly, Vogel et al. [109] utilized a humanized RBL-2H3 cell model to analyze and identify the allergenicity of peanut and hazelnut proteins in complex food matrices.

Furthermore, the Basophil Activation Test (BAT) has gained widespread application for detecting IgE-mediated hypersensitivity reactions [110,111]. This method assesses the expression of activation markers on the surface of basophils and the presence of factors in the supernatant to evaluate allergic reactions. The BAT method has been extensively used in related studies. For instance, Sabato et al. [112] confirmed the high sensitivity of basophils to food allergens through BAT experiments. Additionally, Schwager et al. [90] employed the BAT method to evaluate the allergenicity of natural and recombinant peanut oleosin, finding that roasted peanuts significantly enhanced the allergenicity of this protein.

### 3.6. Animal Model

Animal models play a crucial role in assessing food allergen sensitizing potential. They serve as a bridge between in vitro assessment and real human allergic reactions, and they can closely mimic the human immune response to allergens. By exposing animals to allergens through various routes—such as oral administration, inhalation, or skin contact—similar to human exposure, researchers can observe and measure immune responses and clinical symptoms. The allergenic potential can further be evaluated by detecting specific IgE and IgG antibody levels, as well as assessing allergic inflammation through changes in cytokine profiles. Additionally, animal models provide valuable insights into the mechanisms underlying allergic sensitization and development [56,113]. However, due to inherent differences between animals and humans, results should be interpreted with caution. Appropriate species and strain selection, along with well-designed experiments, are essential to ensure relevance to human biology. Despite these limitations, animal models remain indispensable in allergenicity assessment because they offer in vivo data that closely approximates human allergic reactions [56,113].

Rodents are often the preferred choice for constructing food allergy animal models due to their physiological and genetic similarities to humans [57]. Mice, in particular, are widely used in research because of their unique advantages, including a rich array of genetic resources and high compatibility with genetic manipulations, such as forward genetics, reverse genetics, transgenic technology, and targeted mutations or gene knock-in/knock-out [114]. The extensive application of these genetic engineering techniques in mouse models has led to the development of many genetically modified humanized mouse models, which are valuable tools for studying human diseases. For example, Jimenez-Saiz et al. utilized various mouse strains (e.g., C57BL/6, Rag2-/-γc+, (Igh-7tmlLed) 25, BCL6fl/fl) to elucidate the exposure routes of human food allergens, providing a theoretical basis for potential therapeutic approaches [113]. Additionally, Burton et al. successfully established a humanized adaptive immune system mouse model (huNSG mouse) capable of simulating IgE-mediated food allergic reactions by engrafting human tissue-derived mast cells, thus offering a novel platform for studying IgE-mediated allergic responses [115]. Furthermore, mice have a short breeding cycle (approximately 18–20 days), are cost-effective, and are easy to manage, making them an ideal choice for disease model research [58].

Commonly used experimental rodent strains in food allergenicity evaluation include mouse strains such as C57BL/6, C3H/HeJ, DBA2, and BALB/c, as well as rat strains like BN [57]. Among these, Balb/c mice, which tend to differentiate toward Th2 cells in their immune response, have been extensively utilized in allergy research. For instance, Fotschki et al. [116] sensitized Balb/c mice by intraperitoneal injection and assessed the allergenic potential of food components such as BLG and ALA by measuring specific IgE levels in mouse serum. Wavrin et al. [117] evaluated the allergenic potential of the peanut allergen Arah 1 by exposing Balb/c mice to the allergen through contact, inhalation, and oral administration.

In addition to rodents, other animal models, such as pigs, dogs, and monkeys, are also widely employed for food allergenicity evaluation. These non-rodent models are considered more representative in biomedical research due to their closer genetic relationship with humans and their ability to better simulate human physiological and pathological processes. For example, Li et al. [118] utilized a pig model to evaluate the allergenicity of genetically modified milk containing recombinant human lactoferrin. By monitoring indicators such as the incidence of diarrhea, humoral immune responses, Th2 cell responses, and intestinal mucosal damage, the study demonstrated that the genetically modified milk did not induce allergic reactions. However, despite their similarities to humans in terms of organ size, physiological structure, disease progression, and histological features, the use of these larger mammals in food allergenicity evaluation is limited due to economic and time constraints.

## 4. Conclusions and Perspectives

This review comprehensively clarifies the mechanisms of food allergies and summarizes the methods for assessing food component allergenicity, covering the principles and applications of various techniques. Although these methods differ in their mechanisms, all are developed based on allergen epitope information. By analyzing the residual allergen epitopes and their quantitative and qualitative changes, we can preliminarily predict a component’s allergenic potential.

Future research should develop evaluation methods that better align with food allergy mechanisms. For mass spectrometry, technological advancements are expected to boost detection efficiency and throughput. Meanwhile, more powerful data-processing software and algorithms will aid in the in-depth mining of allergen information, such as modification sites and conformational changes. This will enhance our understanding of allergens’ characteristics and sensitization patterns. In addition, gathering and updating data on allergen amino acid sequences, 3D structures, and antigenic epitopes helps refine existing allergen databases. Integrating various protein properties, such as sequence similarity, domains, and physicochemical characteristics, into advanced predictive algorithms and models can enhance the accuracy and reliability of allergenicity predictions and minimize both false-positive and false-negative rates.

Simulated digestion methods must refine their conditions to better replicate the human digestive environment, factoring in elements like digestive fluid composition and concentration, pH, temperature, and enzyme activity. This will enhance the accuracy of in vitro results in reflecting in vivo conditions. Additionally, integrating simulated digestion with advanced detection techniques such as mass spectrometry and immunoassays allows for real-time monitoring and analysis of allergens and their degradation products as digestion occurs. This approach yields valuable insights into sensitization behavior and allergenicity, establishing a reliable foundation for assessing the digestive stability and allergenic potential of food allergens.

ELISA has high sensitivity and specificity for allergen detection. However, there is still room for improvement. Future research could focus on developing more specific antibodies to minimize cross-reactivity with non-allergenic proteins, thereby reducing false-positive results. Furthermore, the development of multiplex ELISA assays that can simultaneously detect multiple allergens in a single sample is highly anticipated. This would not only improve the efficiency of food allergenicity testing but also reduce costs and resource consumption.

Cell and animal models must be further optimized and standardized to better emulate human immunological and physiological responses, and to expand their application in studying disease mechanisms and evaluating treatments. Additionally, it is also crucial to consider economic and time costs, so that food allergenicity assessments can truly benefit patients, enhance their well-being, and alleviate the health and economic burdens on both patients and society. Looking ahead, the development of more precise, efficient, and cost-effective food allergenicity evaluation methods is anticipated. Such methods would provide stronger scientific support for the diagnosis, treatment, and prevention of food allergies.

## Data Availability

No new data were created or analyzed in this study. Data sharing is not applicable to this article.

## Abbreviation

APCs	Antigen-presenting cells	LAD2	Laboratory of Allergic Diseases 2
BAT	Basophil activation test	LP	Lamina propria
β-Hex	β-hexosaminidase	LTB4	Leukotriene B4
DCs	Dendritic cells	Mono	Monocytes
ELISA	Enzyme-linked immunosorbent assays	MS	Mass spectrometry
Eos	Eosinophils	Neu	Neutrophils
FcεRI	high-affinity IgE receptor	RBL-2H3	Rat Basophilic Leukemia 2H3
GAPs	Goblet cell-associated antigen passages	Th2 cells	T helper 2 cells
GM-CSF	Stimulating factor	Tregs	Regulatory T cells
HMC-1	Human Mast Cell Line 1	TSLP	Thymic stromal lymphopoietin
ILC2s	Lymphoid cells		
